# *Nasonia vitripennis* males exhibit greater effort and competency in detecting hosts with conspecific females than other *Nasonia* males

**DOI:** 10.1098/rsos.251303

**Published:** 2025-11-19

**Authors:** Taruna Verma, Bharat Kumar Sirasva, Satyajit Jena, Diptimayee Behera, Anoop Ambili, Ruchira Sen, Rhitoban Raychoudhury

**Affiliations:** ^1^ Department of Biological Sciences, Indian Institute of Science Education and Research (IISER) Mohali, Knowledge City, Sector-81, Manauli, Punjab 140306, India; ^2^ Department of Physical Sciences, Indian Institute of Science Education and Research (IISER) Mohali, Knowledge City, Sector-81, Manauli, Punjab 140306, India; ^3^ Department of Earth and Environmental Sciences, Indian Institute of Science Education and Research (IISER) Mohali, Knowledge City, Sector-81, Manauli, Punjab 140306, India; ^4^ PG Department of Zoology, Sri Guru Gobind Singh College, Sector 26, Chandigarh 160019, India

**Keywords:** cafeteria assay, mate search, host preference, species recognition, cuticular hydrocarbon profile

## Abstract

*Nasonia* is a species complex of four parasitoid wasps. *N. vitripennis* is cosmopolitan, while the other three species are micro-sympatric with it. This distribution can select distinct species-specific mate recognition capabilities. However, whether *Nasonia* males can distinguish between hosts with conspecific females and those with heterospecific females is not known. Therefore, we test this hypothesis in a cafeteria-based choice assay and show that *N. vitripennis* males can distinguish hosts with conspecific wasps against those parasitized by *N. giraulti* and *N. oneida*, exhibiting longer search time and distance traversed with faster search speed. We also found that *N. longicornis* males can distinguish hosts with conspecific wasps, but only against the hosts parasitized by *N. oneida*. We further investigated the pairwise differences in the cuticular hydrocarbon (CHC) profiles of the parasitized hosts and adult female wasps. The results reveal that males show this ability only when the compounds responsible for differences in adult female CHC profiles are also the key differentiators of the host CHC profiles. The comparative mate searching behaviour of males of all reported species within a genus has rarely been studied. Therefore, this study makes a significant contribution to our understanding of interspecific variation of conspecific-mate searching behaviour.

## 1. Introduction

Successful reproduction requires the timely recognition of conspecific mates. Therefore, finely tuned mate recognition systems have evolved for both sexes in most species [1,2]. Additionally, the reproductive success of males is generally limited by the number of fertile females and the number of eggs laid by them [3]. Thus, sex-specific and divergent selective pressures can lead to the development of distinctive reproductive strategies that maximize fitness [4]. Males can accomplish this directly by intense mate searching [5], multiple mating [3,6], producing pheromones to attract females [7] and attaining protandry or achieving reproductive maturity before the females [8]. Males can also employ several indirect strategies, like male–male competition [9,10], territoriality [11], monopolizing access to females [5], sneaky copulation [12], etc. However, before employing any of these direct or indirect strategies, males must actively search for potential mates by using sensory cues [13] to gain access to as many mates as possible [14]. When females are present in patches, males must also optimize the time and effort spent on each such patch [13,15]. Selection pressure for narrower conspecific mate recognition cues, however, can further intensify if a pool of possible mates contains closely related sympatric species, as speciation is not always accompanied by divergence in species recognition cues [14,16]. This has been shown in many recently diverged taxa where species recognition cues are not distinctive and species-specific [14,16–18].

The parasitoid wasp *Nasonia* (Hymenoptera: Pteromalidae) is one of the most well-characterized insect systems for understanding sexual communication and is an excellent model system for uncovering conspecific mate-finding strategies [19–25]. *Nasonia* is a parasitoid of cyclorrhaphous fly pupae [26] and completes its development inside the host. The protandrous males usually wait on the natal host patch for females to emerge. Females, upon emerging, usually mate only once and fly out to seek fresh pupae to lay eggs [26] by drilling a hole with their ovipositor [27].

Among the four *Nasonia* species, *N. vitripennis* is cosmopolitan and therefore sympatric with the other three species*—N. longicornis* (found in western North America), *N. giraulti* and *N. oneida* (both found in eastern North America) and micro-sympatric with each other across their range [28]. This sympatric distribution can affect the parasitization of a patch of fly pupae by causing multi-parasitism, where the patch can be parasitized by multiple species of *Nasonia* [29–31]. The reproductive interference caused by such multi-parasitism [14,32] can be a selection pressure for the evolution of prezygotic barriers. Therefore, *Nasonia* males should be under selection to evolve precise mate recognition systems [14,32–34]. Evidence indicates that such a sympatric distribution may have given rise to some behavioural phenotypes in *Nasonia*, like the species-specific male courtship behaviour [33] and the evolution of within-host mating, particularly in *N. giraulti* [20,35]. Additionally, the pronounced female-biased sex ratio [26] and avoidance of multiple mating by females [36] can also intensify selection on males to develop precise species-recognition cues. This strategy can include the ability of males to detect hosts with conspecific females and thereby monopolize access to emerging females. Accordingly, Prazapati *et al.* [25] showed that male *Nasonia* can distinguish parasitized hosts from unparasitized ones, and *N. vitripennis* males can even detect which fly hosts have adult females inside them. This ability was found to be correlated with males’ ability to perceive female-specific cuticular hydrocarbons (CHCs) from the hosts [25]. However, this strategy would only work to maximize male fitness if they can detect parasitized hosts with conspecifics. As shown above, theoretically, multiple selection forces should enable *Nasonia* males to distinguish hosts with conspecific mating partners. However, whether such abilities exist has not been studied empirically.

Here, we investigated if *Nasonia* males exhibit any such ability to detect hosts with conspecific wasps by providing them with a choice of hosts with conspecific and heterospecific wasps in a cafeteria assay. Furthermore, we studied the searching behaviour of males by developing a customized Python program for tracking the movement of males and looked for species-specific differences. Finally, we explored the CHC profiles of parasitized hosts and adult females to test the presence of species-specific olfactory signatures that can facilitate the searching behaviour of males.

## 2. Material and methods

### 2.1. Strains used

We conducted all behavioural and CHC profile studies with four *Nasonia* species. We used *Nasonia* strains that were relatively recently collected from the field. The strains used were NV-IPU08 of *N. vitripennis*, NL-MN8501 of *N. longicornis*, NG-RV2XU of *N. giraulti* and NO-NY11/36 of *N. oneida*. All strains were maintained at 24 h constant light, at 25°C temperature with 60% relative humidity. The life cycles of the four species under these conditions were 14 days for *N. vitripennis*, 14.5 days for *N. longicornis*, 15 days for *N. giraulti* and 16 days for *N. oneida*. We obtained virgin males and females by hosting virgin and mated females, respectively, on *Sarcophaga dux* (Diptera: Sarcophagidae) pupae. We obtained parasitized hosts for the cafeteria assay by exposing two hosts (less than 48 h old) to a single mated female in a RIA vial for 48 h. To ensure uniformity and minimize potential variability due to age and prior experience in the behavioural observations, we used virgin males, who were less than 24 h old, and mated and virgin females, who were less than 48 h old.

### 2.2. Cafeteria assay

**Figure 1.**
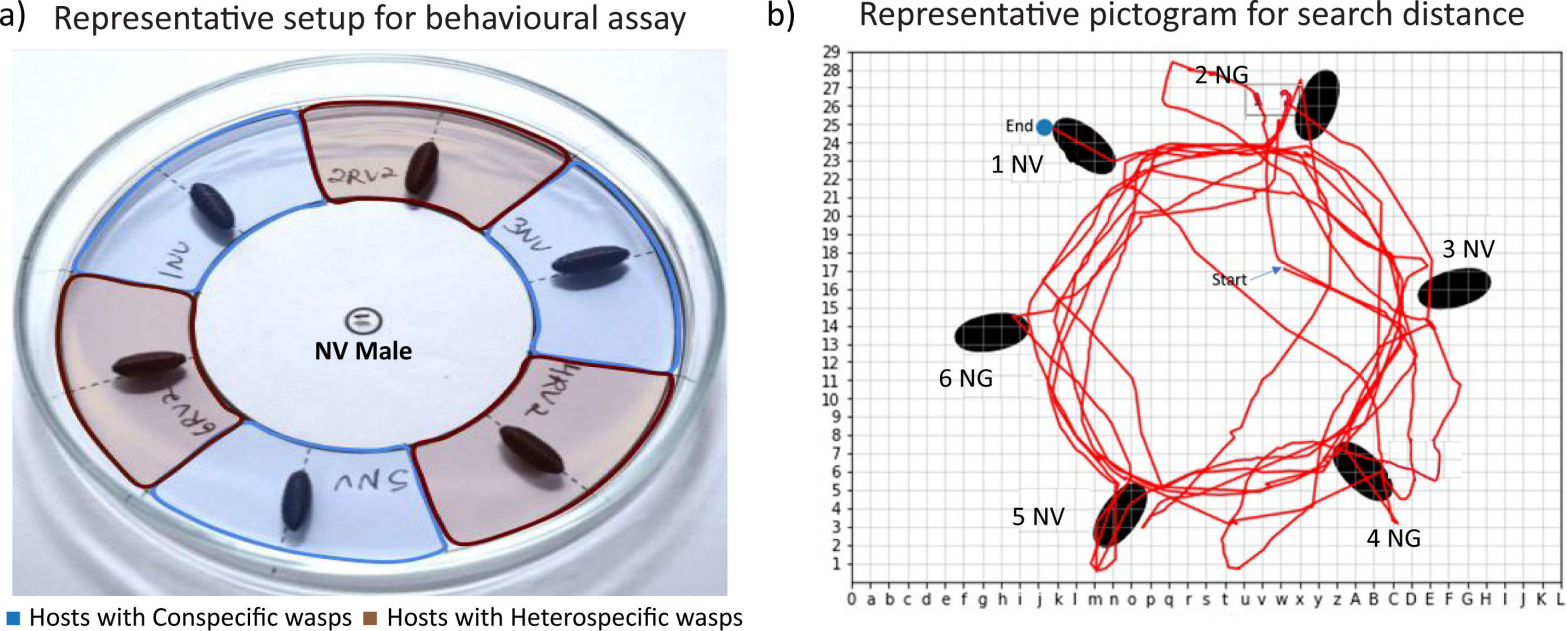
(a) Cafeteria arena setup for the behavioural assays: three hosts parasitized by conspecific females and three hosts parasitized by heterospecific females were placed alternately. In each assay, one male was introduced at the centre of the plate. Video recording was done immediately after introducing the male for 4 min using a Logitech C615 HD webcam. The colours used to distinguish hosts in the picture are only for representation. (b) The distance traversed by the males: movement of each male was tracked and digitally scored by the customized Python program for measuring search distance and calculating search speed.

**Figure 2.**
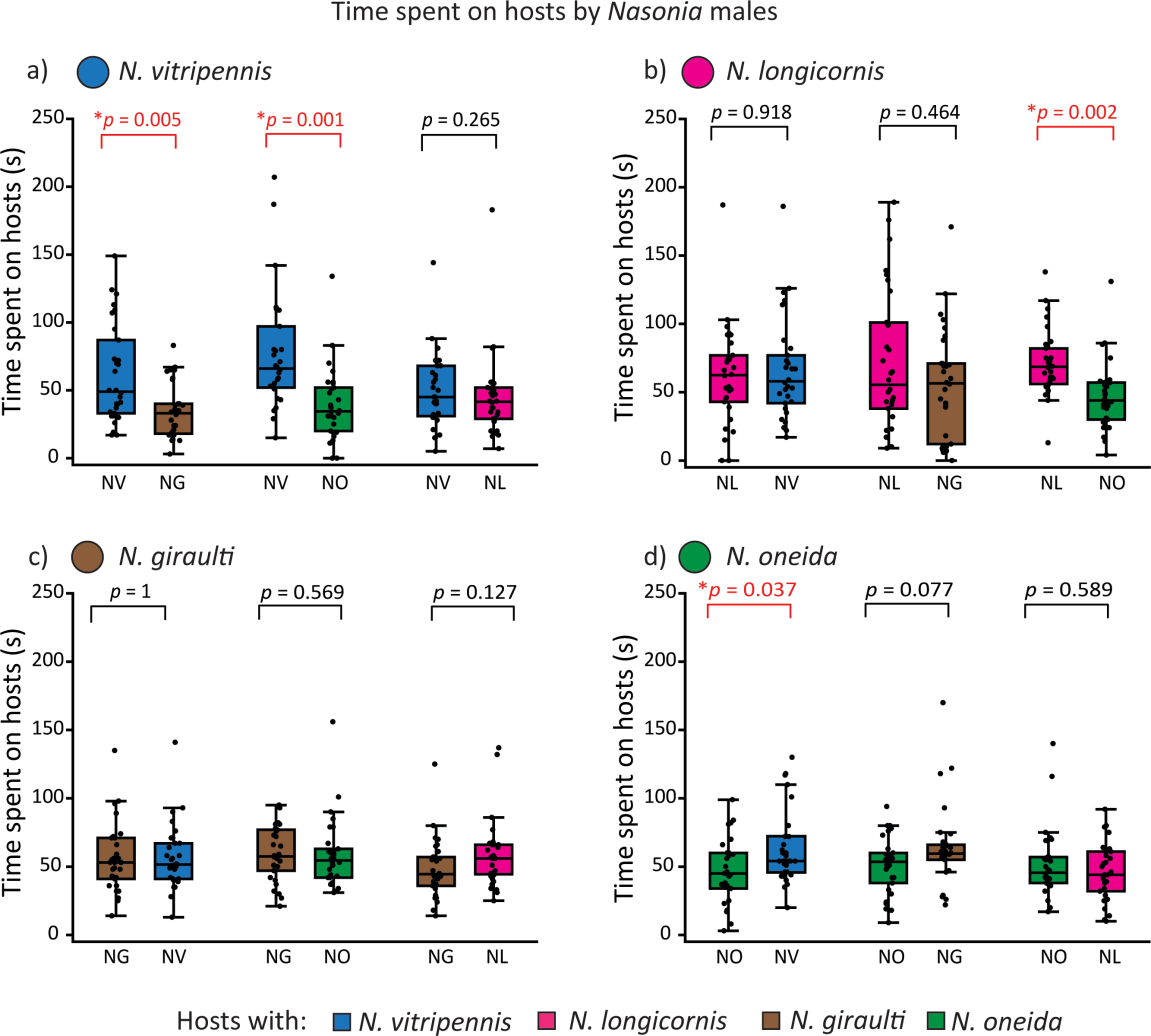
Comparison of wait duration by *Nasonia* males on hosts with conspecific wasps and hosts with heterospecific wasps in the cafeteria assay: (a) *N. vitripennis* males spent significantly more time on hosts with conspecific against hosts with *N. giraulti* and *N. oneida*; (b) *N. longicornis* males spent significantly more time on hosts with conspecific against hosts with *N. oneida*; (c) *N. giraulti* males did not spend significantly longer duration on any host; (d) *N. oneida* males spent significantly more time on hosts with *N. vitripennis* but spent similar time on both kinds of hosts against *N. giraulti* and *N. longicornis*. Each comparison is based on data for 30 assays, and asterisks represent significant differences (Wilcoxon signed-rank test, *p* < 0.05).

We conducted the behavioural assays using an equal choice arena (figure 1a), made of a glass Petri plate (9 cm in diameter), placed on a circular white paper of the same dimension. We drew two concentric circles of 9 and 5 cm in diameter on the paper and divided the annulus into six equal sections [25]. We alternately placed six hosts (three containing conspecific and three with heterospecific wasps) on these six sections. This setup was placed on a wooden platform with a white LED lamp above at a height of 30 cm. To restrict the males within the arena, we made a thin water ring at the circumference of the Petri plate. We then introduced a virgin male in the centre of this arena and recorded its behaviour for the next 4 min using a Logitech C615 HD camera (all data are uploaded at https://www.youtube.com/@Evogeniiserm). We intended to use hosts with mature wasps about to eclose. Therefore, to confirm the parasitization of the hosts with the required pupal stage, we opened each host pupa after the behavioural assay and verified the developmental stage. We discarded the assays where one or more hosts failed to meet this criterion. The males of each species were subjected to three categories of assays involving the other three heterospecific species. For example, each *N. vitripennis* male was placed with three *N. vitripennis* pupae and three *N. longicornis* pupae as the first category, then with three *N. vitripennis* pupae and three *N. giraulti* pupae as the second category and so on. This resulted in 12 different categories (figure 2) of assays across the four species, with 30 data points for each category, where their behaviour was recorded for 4 min. Each data point was collected using a fresh set of Petri plates, hosts and inexperienced virgin males.

To assess whether males can identify hosts with conspecific wasps, we compared the total time each male spent on the two kinds of hosts. The times spent on hosts with conspecific or heterospecific wasps were calculated by summing the total time spent on all three hosts of a similar type. Time spent was recorded from the moment a male climbed on a host and ended when it descended from it. If the males spent significantly more time on hosts with conspecific wasps, we considered them capable of preferring or identifying the hosts with conspecific over hosts with heterospecific wasps.

### 2.3. Searching behaviour

To quantify the different parameters of ‘searching behaviour’, we designed a customized Python program, which tracked each male’s movement in the arena for the entire duration of the behavioural assay (figure 1b; electronic supplementary material, Python script). We incorporated four possible search parameters (table 1) to assess variation in search behaviour: (i) ‘search distance’, (ii) ‘search time’, (iii) ‘search speed’, and (iv) ‘search latency’. For this analysis, we used 24 videos from each category of bioassay and pooled the data for males of a given species across the three choice assays.

**Table 1.** The list of search parameters included in the study to analyse the search behaviour of males and their quantifications.

Search parameter	Definition	Quantification
Search distance	Total distance (in metres) travelled by individual males in the arena for 4 min.	Calculated using a customized program written in Python (electronic supplementary material, Python script), which tracked the movement of males (figure 1b).
Search time	The total duration of time for which males were moving around and not stationed on a particular host.	Calculated by subtracting the time spent on hosts from the total assay time (4 min).
Search speed	The average speed at which males were moving in the cafeteria arena.	Calculated by dividing the search distance by the search time in m s^−1^
Search latency	The time taken by a male (in seconds) to find the first host with conspecifics. Finding was scored when a male climbed on a host.	Calculated as the time from the start of the assay until the male found the first host with conspecifics.

### 2.4. Purification and identification of cuticular hydrocarbons

We analysed the CHCs of the parasitized hosts and the adult virgin females of all four species. For these eight categories, we had three replicates, each prepared from 50 individual hosts or 50 females.

We collected CHCs from parasitized hosts (13 to 15 days old, depending on the *Nasonia* species) by dipping them in 3 ml of *n*-hexane (HPLC grade, Merck) for 5 min. The acquired solution was then concentrated to 100 µl under a gentle nitrogen stream. The process was repeated for adult females, which were similarly dipped in 500 µl of *n*-hexane for 10 min. The collected extracts were then processed to concentrate the CHCs using column chromatography in glass columns (0.7 cm inner diameter), which were packed 3 cm high with baked glass wool and silica gel. Eluted fractions of hosts and females were then separately concentrated to a 50 μl volume in a gentle nitrogen stream.

To identify the chemical signature of these extracts, the samples were run through a gas chromatograph (GC) connected with a mass spectrometer (Agilent 7890B, 5977C GC-MS) with an HP-5MS (Agilent J&W) capillary column (30 m × 0.25 mm × 0.25 µm), operated in a 70 eV electron impact ionization mode. The quadrupole temperature was maintained at 150°C, and the inlet and auxiliary line temperatures were kept at 320°C. Two microlitres (1 host or female equivalent per µl) of the extracted sample were injected individually in a split-less mode into the GC machine, where helium was used as the carrier gas at an average linear velocity of 36.2 cm s^−1^. Separation of these fractions was advanced, keeping the initial temperature at 40°C, which was held for 5 min. The oven temperature was then raised to 320°C.

The percentage of the relative abundances was calculated for each detected compound (electronic supplementary material, tables S9 and S10). The identification of the compounds involved a comparison of the distinctive diagnostic ions present in the obtained mass spectra with the NIST library of Mass Hunter Workstation Software v. B.08.00 (Agilent Technologies) and *n*-alkane standards (*n*-C_8_ to *n*-C_40_; Sigma-40147-U [25]). Methyl-branched hydrocarbons were identified through their characteristic diagnostic ions arising from the fragmentations at their branching points and the utilization of linear retention index values from previously published data [22,25,37].

To identify the potential CHC compounds responsible for host recognition by the males, we parsed the CHC profiles from hosts parasitized by each species of females. The full GC profiles and their corresponding SIMPER analyses are given in electronic supplementary material, tables S11–S15. We focused on the unique peaks as well as peaks with varying relative abundances [38], which together contributed to 50% of the total dissimilarity between these profiles [39]. This was also done for the adult female profiles (the full GC profiles and their corresponding SIMPER analyses are given in electronic supplementary material, tables S16–S20), as hosts contain more females than males due to the female-biased sex ratio of *Nasonia* [26].

## 3. Statistical analyses

We assessed the data distribution using the Shapiro–Wilk test for normality [40] and used parametric and non-parametric tests for normally distributed data and non-normally distributed data, respectively. We employed the Wilcoxon signed-rank test for the time spent on hosts by males. We used one-way ANOVA, followed by Tukey’s HSD *post hoc* test, to compare interspecific differences in search time and speed. In contrast, for the analysis of the other two search parameters, search distance and search latency, we used the Kruskal–Wallis test, followed by Dunn’s *post hoc* test. We conducted principal components analysis (PCA) on the CHC profiles. Prior to carrying out the PCA, we added a small constant to the dataset before transformation to address the presence of undetectable compounds in some samples [41] and then transformed the data using centred log-ratio [42]. We identified the CHCs responsible for contributing to species-specific differences by a SIMPER analysis, which ranks the contribution of individual CHC peaks based on relative abundance and presence and/or absence of the compounds by Bray–Curtis distance measures [43] using Past4.03.exe [44]. We used R v. 4.2.2 [45] for data transformation and Origin 2022 [46] for all statistical analysis and constructing the box plots and bar graphs.

## 4. Results

### 4.1. *Nasonia vitripennis* and *Nasonia longicornis* males can identify hosts with conspecific wasps

We found a distinct pattern of host preference in the choice assays, where males of all four *Nasonia* species were placed with hosts with conspecific and heterospecific wasps (figure 2; electronic supplementary material, tables S1–S4). Males of *N. vitripennis* spent significantly more time on the host with conspecifics against *N. giraulti* and *N. oneida* (Wilcoxon signed-rank test, *n* = 30, *p* = 0.005 and *n* = 30, *p* = 0.001, respectively; figure 2a). However, they did not show any preference for hosts with conspecifics against hosts parasitized by *N. longicornis* and spent nearly equal time on both (Wilcoxon signed-rank test, *n* = 30, *p* = 0.265; figure 2a). This indicates that *N. vitripennis* males can identify hosts with conspecifics from hosts with heterospecifics, except when the latter is parasitized by *N. longicornis*. Males of *N. longicornis* spent significantly more time on hosts with conspecifics only against *N. oneida* (Wilcoxon signed-rank test, *n* = 30, *p* = 0.002; figure 2b), but did not show any preference to hosts with conspecifics against hosts parasitized by *N. vitripennis* and *N. giraulti* (Wilcoxon signed-rank test, *n* = 30, *p* = 0.918 and *n* = 30, *p* = 0.464, respectively; figure 2b). Males of *N. giraulti* did not exhibit any preference in time spent on hosts with conspecifics over hosts parasitized by *N. vitripennis*, *N. oneida* and *N. longicornis* females (Wilcoxon signed-rank test, *n* = 30, *p* = 1; *n* = 30, *p* = 0.569; and *n* = 30, *p* = 0.127, respectively; figure 2c). Similarly, males of *N. oneida* failed to distinguish between hosts with conspecific and those parasitized by *N. giraulti* and *N. longicornis* (Wilcoxon signed-rank test, *n* = 30, *p* = 0.077 and *n* = 30, *p* = 0.589, respectively; figure 2d). Interestingly, *N. oneida* males spent significantly more time on hosts parasitized by *N. vitripennis* females (Wilcoxon signed-rank test, *n* = 30, *p* = 0.037; figure 2d) than on hosts parasitized by conspecific females.

### 4.2. *Nasonia vitripennis* and *Nasonia longicornis* males use different strategies while searching

As the above results indicate, *Nasonia* males have varying abilities to identify hosts with conspecifics. To understand whether this varying ability is an outcome of how these males search for conspecifics, we compared the search behaviour of four species by exploring four parameters: search distance, search time, search speed and latency to find the first host with conspecifics (search latency) (table 1).

**Figure 3.**
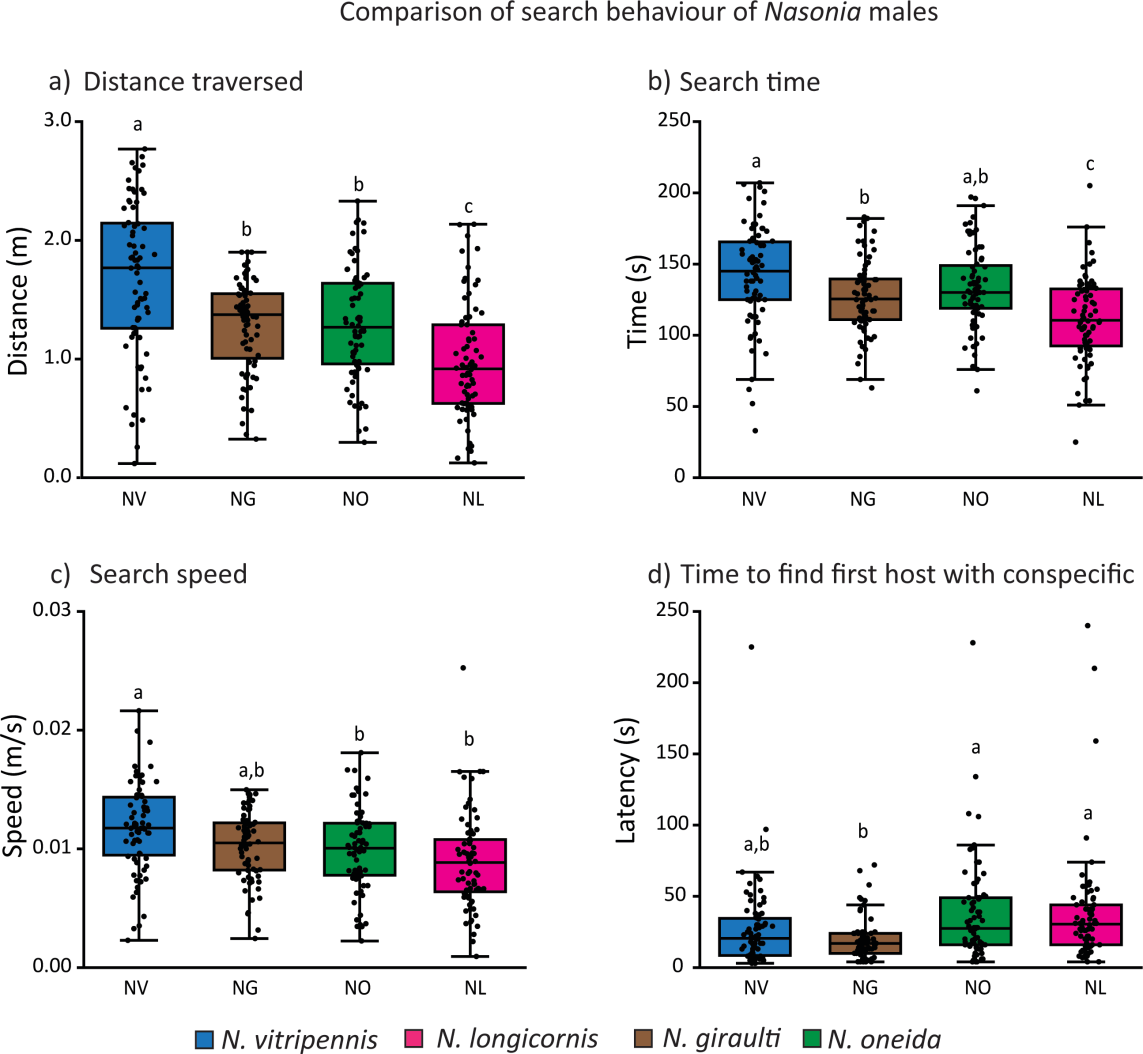
Comparison of four parameters of search behaviour of *Nasonia* males: (a) search distance, (b) search time, (c) search speed and (d) search latency. Boxes carrying different alphabets represent significant differences. (Kruskal–Wallis with Dunn’s as *post hoc* test, *p* < 0.05 for search distance and latency, and one-way ANOVA with Tukey’s HSD *post hoc* test, *p* < 0.05 for search time and search speed.) For each species, 72 data points were used, and based on the data distribution, parametric or non-parametric tests were employed.

We compared the distance traversed (search distance) by males in 4 min and found that *N. vitripennis* males cover the longest distance, which was significantly more than males of the other three species (Kruskal–Wallis with Dunn’s test *n* = 72, *p* = 0.004, *n* = 72, *p* = 0.003, *n* = 72, *p* < 0.0001 with *N. giraulti*, *N. oneida*, *N. longicornis*, respectively; figure 3a; electronic supplementary material, table S5). There was no significant difference in the distances covered by *N. giraulti* and *N. oneida* (Kruskal–Wallis with Dunn’s test, *n* = 72, *p* > 0.05; figure 3a; electronic supplementary material, table S5), while *N. longicornis* covered the least distance (Kruskal–Wallis with Dunn’s test, *n* = 72, *p* = 0.001 and *n* = 72, *p =* 0.001 for *N. giraulti* and *N. oneida*, respectively; figure 3a; electronic supplementary material, table S5). Therefore, the success of *N. vitripennis* in being the most successful species in identifying conspecific mates could be due to the effort that each male spends in covering the longest search distance.

As we compared the search time of the four species, we found that *N. vitripennis* males also spent the most time searching for hosts, which was significantly longer than that of *N. giraulti* but not significantly different from that of *N. oneida* males (one-way ANOVA with Tukey’s HSD, *n* = 72, *p* = 0.027 and *n* = 72, *p* = 0.364 for *N. giraulti* and *N. oneida,* respectively; figure 3b; electronic supplementary material, table S6). There was no significant difference in search time between *N. giraulti* and *N. oneida* males (one-way ANOVA with Tukey’s HSD, *n* = 72, *p* = 0.63705; figure 3b; electronic supplementary material, table S6). However, the search time for all three species was significantly longer than that of *N. longicornis* males (one-way ANOVA with Tukey’s HSD, *n* = 72, *p* < 0.001, *n* = 72, *p* = 0.010, *n* = 72, *p* < 0.001 for *N. vitripennis*, *N. giraulti*, *N. oneida*, respectively; figure 3b; electronic supplementary material, table S6).

We further compared the search speed (a function of the above two parameters) of the males. Males of *N. vitripennis* exhibited the fastest search speed (electronic supplementary material, table S7), which was significantly different from that of *N. longicornis* and *N. oneida* males but not from that of *N. giraulti* males (one-way ANOVA with Tukey’s HSD, *n* = 72, *p* < 0.001, *n* = 72, *p* = 0.013 and *n* = 72, *p* = 0.056 for *N. longicornis*, *N. oneida* and *N. giraulti*, respectively; figure 3c). However, the search speed of the males of the other three species was not significantly different from each other (one-way ANOVA with Tukey’s HSD, *n* = 72, *p* > 0.05; figure 3c; electronic supplementary material, table S7).

Although *N. vitripennis* males significantly differed from the other species in the above three parameters, they did not differ in the latency to find the first host with conspecific wasps (Kruskal–Wallis with Dunn’s test, *n* = 72, *p* > 0.05; figure 3d). *N. giraulti* exhibited a shorter latency than *N. longicornis* and *N. oneida* (Kruskal–Wallis with Dunn’s test, *n* = 72, *p* < 0.001 and *n* = 72, *p* < 0.001 for *N. longicornis* and *N. oneida*, respectively; figure 3d; electronic supplementary material, table S8), but it was not significantly different from *N. vitripennis* (Kruskal–Wallis with Dunn’s test, *n* = 72, *p* = 0.390; figure 3d; electronic supplementary material, table S8).

### 4.3. Comparative analysis of olfactory cues

**Figure 4.**
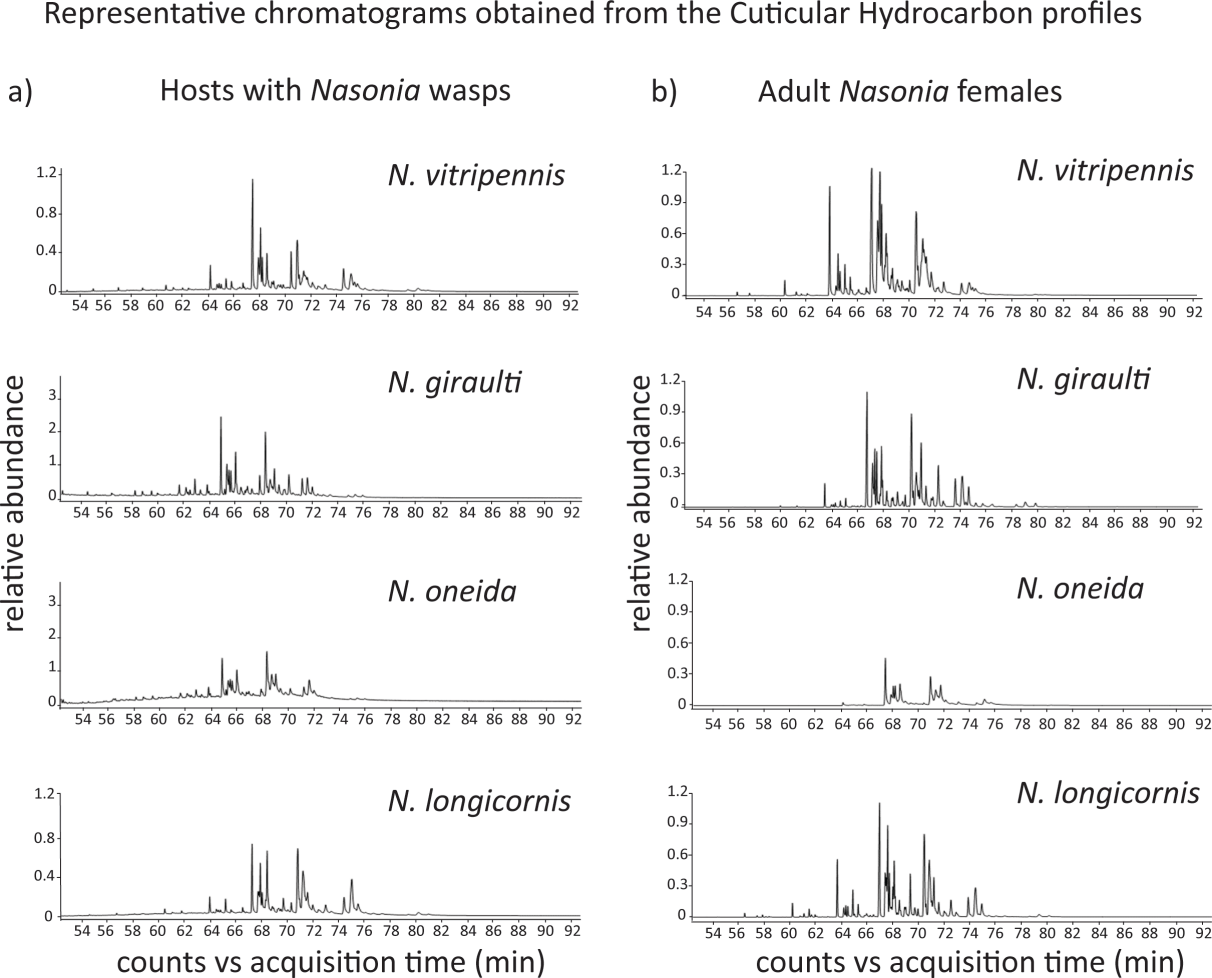
(a) Representative chromatograms obtained from the CHC profiles of hosts with adult males and females inside. A total of 46 peaks were identified in CHC profiles of hosts parasitized by *Nasonia* females of different species. (b) Representative chromatograms obtained from the CHC profiles of adult *Nasonia* females. A total of 50 peaks were identified in CHC profiles of *Nasonia* females of different species.

The GC-MS analysis of the CHC profile of the parasitized hosts (having adults inside) and adult females shows the presence of long-chain saturated and unsaturated hydrocarbons ranging from *n*-C_25_ to *n*-C_37_ (figure 4; electronic supplementary material, tables S9 and S10, respectively), with the majority composed of methyl-branched alkanes. Forty-six peaks were identified in host CHC profiles, out of which 43, 34, 33 and 38 peaks were found in hosts parasitized by *N. vitripennis*, *N. giraulti*, *N. oneida* and *N. longicornis* females, respectively (electronic supplementary material, table S9). In contrast, 50 peaks were identified in CHC profiles of adult females with four additional peaks: MeC25(5-), MeC27(7-), MeC27(5-) and MeC28(4-). Specifically, 48, 48, 29 and 45 peaks were found in *N. vitripennis*, *N. giraulti*, *N. oneida* and *N. longicornis* females, respectively (electronic supplementary material, table S10). The identified peaks in the female CHC profile contain both previously reported [47] and newly identified peaks.

*Nasonia* males can utilize olfactory cues in the form of female CHCs to distinguish different types of hosts [25]. In the choice assays of this study, we found that *N. vitripennis* males can detect the hosts with conspecific wasps against hosts with *N. giraulti* and *N. oneida*, and *N. longicornis* males can achieve this against hosts with *N. oneida.* To understand the potential olfactory cues responsible for the identification, we compared the CHC profiles of every combination of hosts offered in the choice assays to these two species. Furthermore, to investigate if the host CHCs had any similarity with the CHCs of the females within, we further compared the CHC combinations of females.

**Figure 5.**
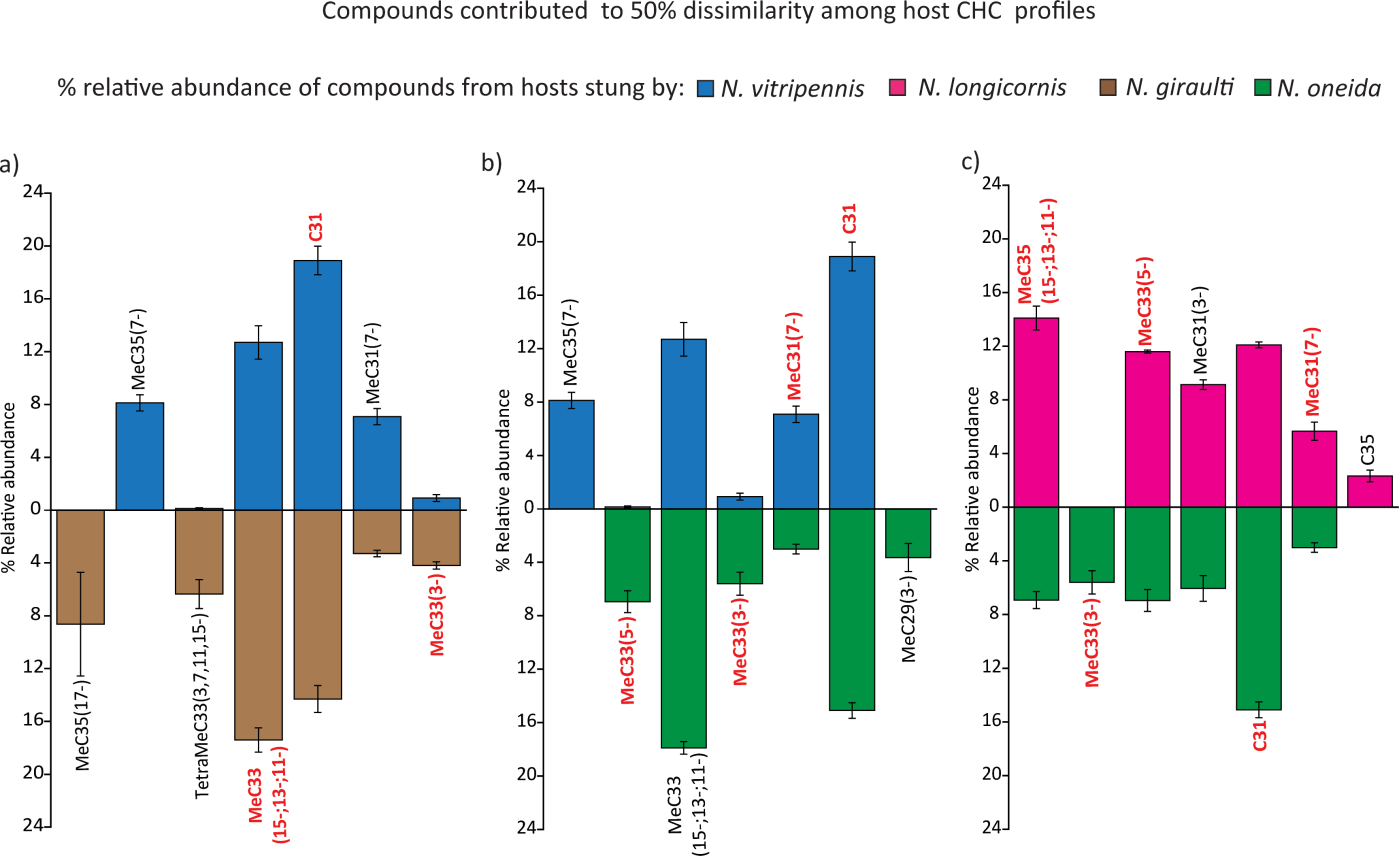
Relative abundance (%) of compounds that cumulatively contributed to 50% of dissimilarity between CHC profiles of parasitized hosts, which were distinguished by males. Comparison of compounds between (a) hosts parasitized by *N. vitripennis* and *N. giraulti,* (b) *N. vitripennis* and *N. oneida,* (c) hosts parasitized by *N. longicornis* and *N. oneida*. Compounds titled in red also contribute to dissimilarity in adult female CHC profiles of the corresponding species.

Seven different peaks contributed to 50% of the dissimilarity observed between the hosts parasitized by *N. vitripennis* and *N. giraulti* (figure 5a; electronic supplementary material, table S11). Out of these, three peaks (C31, MeC33(3-) and MeC33(15-;13-;11-)) were also found to contribute to 50% of the dissimilarity seen in the adult female profiles of these two species (electronic supplementary material, table S16). Similarly, the comparison between hosts parasitized by *N. vitripennis* and *N. oneida* also yielded seven such peaks (figure 5b; electronic supplementary material, table S12). Out of these, four peaks (C31, MeC33(3-), MeC31(7-) and MeC33(5-)) contributed to 50% dissimilarity of adult females of these two species (electronic supplementary material, table S17). These results suggest that in cases of successful identification, the main compounds responsible for the dissimilarity in females can also contribute to the olfactory uniqueness of the hosts. This contention is further strengthened by the comparisons with indistinguishable hosts parasitized by *N. vitripennis* and *N. longicornis*, where six peaks contributed to 50% dissimilarity (electronic supplementary material, figure S1a, table S13), but only one of them, MeC35(15-;13-;11-), was common to those contributing to 50% dissimilarity between the females of corresponding species (electronic supplementary material, table S18).

*N. longicornis* males were able to identify the hosts with conspecific wasps only against hosts parasitized by *N. oneida.* The CHC comparison of hosts parasitized by these two species revealed seven peaks contributing to 50% dissimilarity of the hosts (figure 5c; electronic supplementary material, table S14). Among them, five peaks (C31, MeC33(3-), MeC33(5-), MeC31(7-) and MeC35(15-;13-;11-)), were also found to be different between the adult females of these two species (electronic supplementary material, table S19). Comparison between hosts parasitized by *N. longicornis* and *N. giraulti* showed five peaks (electronic supplementary material, figure S1b; SIMPER analyses, electronic supplementary material, table S15) to be different, but shared only one, MeC33(3-), with the adult female profiles (electronic supplementary material, table S20).

These results indicate that successful identification by *N. vitripennis* and *N. longicornis* males may require the presence of a blend of female-specific CHCs in the host, as when these peaks were absent, the males failed to identify hosts with conspecifics.

**Figure 6.**
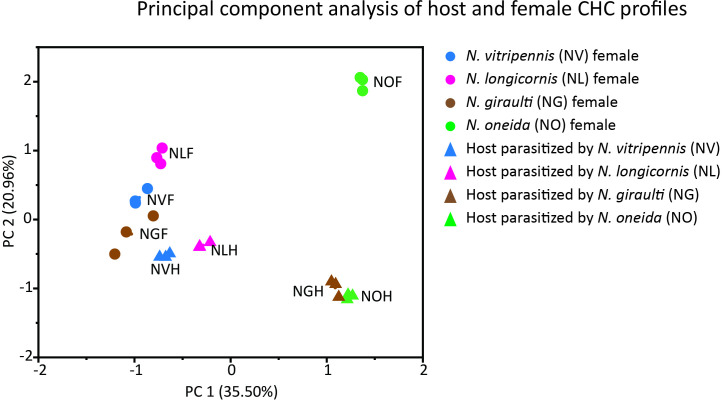
Distribution of CHC profiles of *Nasonia* females (circles) and parasitized hosts (triangles) in two-dimensional principal components space.

The above results aligned with the PCA of the relative abundance (%) for the CHCs extracted from the parasitized hosts and females of four species (figure 6). The first two principal components explained 56.46% variation, and when plotted against each other, separations were seen among the CHC profiles of parasitized hosts and females (figure 6). The hosts parasitized by *N. vitripennis* and *N. longicornis* were clustered together and away from the cluster of hosts parasitized by *N. giraulti* and *N. oneida.* Furthermore, the profiles of hosts parasitized by *N. vitripennis* and *N. longicornis* were close to the profiles of their respective females, but the hosts parasitized by *N. giraulti* and *N. oneida* were relatively far from their respective females.

## 5. Discussion

*Nasonia* males can mate with numerous females in quick succession, limited only by the availability of females [48]. The females emerge from hosts (fly pupae), which usually occur in batches, and can be parasitized by more than one species of *Nasonia* [29–31]. Therefore, to maximize the number of successful matings, a male must find hosts that are parasitized by conspecific wasps and wait for the females to emerge. Moreover, the emerging *Nasonia* females mate only once before flying away to look for fresh hosts to parasitize [26,49,50]. Therefore, the males should be under selection to optimize their energy expenditure and search time to locate hosts with conspecific females and wait. We expected that (i) males should be able to identify the hosts with conspecific wasps and exhibit that by spending more time on them and (ii) they should optimize the duration of waiting on a host and energy expenditure in terms of distance traversed and speed of search. To test these hypotheses, we placed three hosts with conspecific wasps and three hosts with heterospecific wasps in a cafeteria assay. We considered a longer wait (time spent) on the hosts with conspecific wasps to be a measure of the males’ ability to identify the hosts with conspecific wasps. We observed that *Nasonia* males could identify hosts with conspecific wasps, but this ability was not uniform across the species or even within each species. *N. vitripennis* showed this ability against *N. oneida* and *N. giraulti* but not against *N. longicornis*. Whereas *N. longicornis* showed this ability only against *N. oneida*, *N. giraulti* and *N. oneida* did not exhibit any recognition of hosts with conspecifics (figure 2). Moreover, our results also indicated that among the two species that had this capability, the pattern of searching was noticeably different (figure 3). *N. vitripennis* searched for longer durations, travelling the longest distance and at the quickest speed, while *N. longicornis* males traversed the shortest distance with the slowest speed and searched for shorter durations than *N. vitripennis*. These results indicate that recognition of hosts with conspecific wasps can be achieved by both strategies in terms of energy expenditure.

To corroborate the role of olfactory cues in the asymmetry of recognition ability of the four species of *Nasonia*, we explored the CHC profiles of the parasitized hosts and females of *Nasonia*. It is well established that, except for *N. giraulti*, *Nasonia* males can identify and respond to female-derived CHCs [22,47,51,52]*.* It has also been suggested that males likely perceive the cues left by females while ovipositing and/or emanating from the adult females developing inside the host for identification [25]. The porous structure of the fly puparium [53] can also facilitate males in perceiving the olfactory cues from females within. The volatility of the CHC compounds depends on carbon chain lengths, which is inversely proportional to the degree of volatility [54]. The compounds present on both the hosts and the females had long carbon chains (electronic supplementary material, tables S9 and S10), which indicated low volatility. Therefore, we conjecture that the cumulative cues from the low-volatility compounds within the host may provide a perceivable signal for the males. Our results suggest that successful identification corresponds to the compounds that contribute to the maximum difference in CHC profiles of hosts, which are also present in the female profiles. This indicates that males probably identify hosts with conspecifics by identifying the cues derived from adult females still inside the host. The effectiveness of these low-volatility CHCs serving as the cue is also consistent with the males’ inability to fly and being confined to their natal patch. Further empirical validation by isolating and testing these compounds is required to understand the nature of CHC utilization by males.

The asymmetric and species-specific nature of the recognition appears to result from the probability of encountering heterospecific species based on the geographical distribution of the four species. The sympatric distribution can lead to selection for enhanced species-specific ability to distinguish mates [55–60]. Accordingly, *N. vitripennis*, being cosmopolitan and sympatric with the other three species [28], shows the most extensive ability. However, this biogeographic argument is inconsistent with its inability to distinguish the sympatric *N. longicornis*. Moreover, *N. vitripennis* and *N. longicornis* showed a reciprocal inability to distinguish each other’s hosts with conspecific wasps. The other possibility could be a strain-specific effect. The *N. vitripennis* strain used (NV-IPU08) in this study is an Indian strain, which probably does not encounter any heterospecifics, as there is no report of any other species of *Nasonia* from India. However, this cannot explain why *N. vitripennis* can distinguish conspecifics against hosts stung by *N. giraulti* and *N. oneida*. But future studies should use *N. vitripennis* field strains from western North America, which are truly sympatric with *N. longicornis*, to understand whether such sympatric distribution can be correlated with this ability. This biogeographic explanation is also consistent with *N. longicornis’* inability to distinguish hosts with conspecific wasps over hosts parasitized by the allopatric *N. giraulti.* However, it can distinguish hosts with conspecific wasps from hosts parasitized by *N. oneida*, which is largely distributed across eastern North America and is allopatric to *N. longicornis*. The distribution of *N. oneida*, although concentrated in eastern North America, can extend to the Midwest until Lake Mendota (Wisconsin, USA, Raychoudhury and Werren, personal observation). This indicates that *N. longicornis* and *N. oneida* can encounter each other in their native ranges and, therefore, can develop species-specific cues for the identification of hosts with conspecific wasps.

*N. giraulti* shows very high rates of within-host mating [20,35]. Since female *Nasonia* usually mate only once [36], there are substantially fewer opportunities to mate with *N. giraulti* females outside the host. This indicates *N. giraulti* males should not be under selection to identify hosts with conspecific wasps. Our results affirm this prediction. Conversely, the low incidence of within-host mating in *N. vitripennis* and *N. longicornis* [20,35] can be a strong selection pressure to correctly identify mates, which can explain why these are the only two species that can distinguish hosts with conspecifics. Moreover, both these species can show territorial and aggressive behaviour towards other males [19,61,62], indicating that they can be territorial in guarding hosts with females within them. However, both the biogeographic and within-host mating arguments cannot explain why *N. oneida* does not show this capability, as it is sympatric to both *N. vitripennis* and *N. giraulti* [28] as well as exhibits intermediate rates of within-host mating [35]. One possibility could be the loss of this ability in the most recent common ancestor of *N. oneida* and *N. giraulti*, which remains to be verified. However, *N. oneida* females exhibit the strongest mate preference [28] for conspecifics among all the *Nasonia* species. This can result in reduced selection pressure on the males to identify hosts with conspecific wasps, as the females will rarely mate with any heterospecific males. But why *N. oneida* males would prefer hosts parasitized by *N. vitripennis* females (figure 2d) is difficult to explain. Such paradoxical behaviour by *N. oneida* males has also been documented in a previous study [59], where they reject conspecific females at nearly the same rate as heterospecific ones.

*Nasonia* is one of the best-characterized insect model systems to study sexual communication [19–24]. However, as *Nasonia* is also a flagship genus for studying local mate competition and sex ratio variations, most studies have focused only on females [52,63–67]. Recent studies [25,61,62,68,69] have also revealed the complexities of male behaviour. This study unveiled a much more varied and complex reproductive behavioural repertoire of *Nasonia* males than previously known.

## Data Availability

All behavioural assay videos are available at https://www.youtube.com/@Evogeniiserm. Python script used to track the movement of males and other data are given in the electronic supplementary material [70].

## Authors' Notes

All authors agreed to the submission of the manuscript, and R.R. has been authorized as the corresponding author by all co-authors.
